# The effectiveness of a telephone smoking cessation program in mental health clinic patients by level of mental well-being and functioning: a secondary data analysis of a randomized clinical trial

**DOI:** 10.1186/s12889-023-16975-z

**Published:** 2023-11-07

**Authors:** Sarah Swong, Andrew Nicholson, David Smelson, Erin S. Rogers, Omar El-Shahawy, Scott E. Sherman

**Affiliations:** 1 New York University Grossman School of Medicine, New York, NY 10016-6402 USA; 2 Department of Population Health, New York University Grossman School of Medicine, New York, NY USA; 3 University of Massachusetts School of Medicine, Worcester, MA USA; 4 Department of Medicine, VA New York Harbor Healthcare System, New York, NY USA

**Keywords:** Smoking cessation, Telephone counseling, Mental health

## Abstract

**Background:**

Few studies have examined the effectiveness of telephone smoking cessation interventions by severity of behavioral health symptoms. Using data from a telephone counseling study, we examined whether abstinence rates varied by level of behavioral health symptoms.

**Methods:**

The parent study recruited adults who smoke cigarettes (*N* = 577) referred by mental health providers at six Veterans Health Administration facilities. Participants were randomized to specialized telephone counseling (intervention) or state Quitline referral (control). Participants completed assessments at baseline and 6 months, including the BASIS-24, a self-report measure of behavioral health symptoms and functioning. We used the BASIS-24 median to dichotomize participants as having high or low scores. The primary outcome was 30-day self-reported abstinence at 6 months. We compared groups on outcomes by logistic regression and performed an interaction effect analysis between treatment assignment and groups.

**Results:**

At baseline, those with high behavioral health symptoms scores reported heavier nicotine dependence and more sedative and/or antidepressant use, compared to participants with low behavioral health symptoms. At 6 months, participants with low behavioral health symptoms scores in the intervention reported higher rates of 30-day abstinence compared to those in the control arm (26% vs 13%, OR = 2.3, 95% CI = 1.8, 2.9). People with high behavioral health symptoms scores reported no difference in 30-day abstinence between the treatment assignments at 6 months (12% vs. 13%, OR = 1.1, 95% CI = 0.6, 2.0).

**Conclusions:**

Only participants with low behavioral health symptoms scores reported higher abstinence rates in the intervention compared to the state Quitline. Future research can examine alternative approaches for people with worse mental well-being and functioning.

**Trial registration:**

The parent study is registered at www.clinicaltrials.gov NCT00724308.

## Introduction

Smoking remains a leading cause of preventable death. People with mental illness smoke 40% of all cigarettes in the United States and experience excess smoking-related mortality and morbidity [1, 2]. Observational research has established that people with serious mental health problems, including those with serious mental illness (SMI, e.g. psychotic disorders, schizophrenia, and bipolar disorder) and people with severe psychological or mental distress, are more likely to smoke cigarettes, thus making these patients an important target for cessation interventions [3–7]. Heavier nicotine dependence [8], lower distress tolerance [9], and acute psychological distress reactivity [10] have been proposed mechanisms underlying difficulty quitting and higher rates of relapse among people with mental illness who smoke.

Contrary to prevalent beliefs among health professionals, people with mental health disorders can and are motivated to quit [11]. Cessation likely does not negatively impact mental health outcomes and may in fact improve psychological symptoms [12, 13]. Thus, determining effective cessation interventions for people who smoke with worse mental well-being and functioning should be a high priority for clinicians and public health researchers.

Evidence-based interventions, including counseling and cessation medications, are effective in the general population as well as for people with serious mental illness, though less evidence on the efficacy of these interventions exists for people with reported psychological or mental distress [14–16]. Proactive approaches to recruitment and telephone counseling in particular are effective in people who smoke with post-traumatic stress disorder (PTSD) and those with SMI [12, 17–19]. However, the question remains whether the characteristics of the telephone treatment received may impact abstinence outcomes differently depending on the extent of a person’s reported mental well-being and functioning. Are all forms of telephone counseling equally effective for patients with mental health diagnoses? This secondary data analysis is based on a randomized clinical trial comparing an intensive telephone care coordination program to state Quitline counseling among veterans who smoke and receive mental health services at Veterans Health Administration (VA) facilities. This paper examines whether abstinence outcomes in this population differed by Behavior and Symptom Identification Scale (BASIS-24) scores, which measure levels of mental well-being and functioning among populations in mental health clinics, as well as others [20].

## Methods

The TeleQuit Mental Health study tested a specialized, multi-session telephone smoking cessation program for mental health patients who smoke (*N*= 577) referred by mental health providers at six VA facilities in the Northeast [21, 22]. Any patient seen in the mental health clinics was eligible for referral via electronic medical record (EMR) consult, a 10–15 s process linked to a tobacco use clinical reminder. Providers were encouraged to refer all people who smoke regardless of desire to quit. Other eligibility criteria included having access to a telephone, having a mailing address, and having smoked cigarettes in the past 30 days. Referred patients were mailed a welcome packet with information about the study. Study staff called patients up to 5 times to screen and enroll participants. Participants were randomized to receive either a) multi-session (up to 9 calls) telephone cessation counseling designed for patients with a mental health diagnosis or b) warm transfer via three-way call to the Quitline in their state. All participants were offered cessation medications (nicotine replacement therapy [NRT] or bupropion) and mailed a self-help educational packet.

The intervention group was randomized to receive counseling from a VA counselor trained to work with people who have a mental health diagnosis. The VA counselors followed a proactive counseling and relapse-sensitive scheduling protocol created specifically for the study. The content of sessions was based on motivational interviewing and problem-solving therapy that addressed topics such as motivation, comorbid mental health symptoms, coping strategies, medication usage, and relapse prevention. The counselors were trained to proactively discuss the relationship between mental health symptoms and smoking, and to help participants identify mental health-related smoking triggers and coping strategies. The VA counselors also provided ongoing coordination of smoking cessation medications from a VA prescriber and placed notes in the medical record alerting each patient’s referring mental health provider about the patient’s smoking cessation progress. VA counseling involved pre-quit planning (1–6 sessions, 30–60 min each) and post-quit follow-up sessions that followed a manual for consistency and fidelity monitoring (0, 1, 3, 7, 14, 21, and 30 days after their quit date at 10–15 min per session). By comparison, participants randomized to state Quitline counseling received a “warm transfer” to their state Quitline via a three-way call initiated by a research assistant to begin the counseling process. After the three-way call, study staff were not involved in Quitline counseling, which offered standard service that varied state-to-state in terms of the typical number of counseling sessions (ranging from 1 to 6), typical length of first session (ranging from 20 to 45 min), and typical length of follow-up sessions (ranging from 10 to 20 min), as described in the protocol paper for the randomized control trial [22].

Participants completed telephone assessments at baseline, 2 months, and 6 months. Behavioral health symptoms and functioning were measured at baseline using BASIS-24, a 24-item measure that has been validated across race/ethnicity, in outpatient settings, and in patients with schizophrenia [23–26]. BASIS-24 is not a direct measure of either serious mental illness or psychological/mental distress. BASIS-24 is a measure of mental well-being and functioning among populations in mental health clinics. It broadly measures behavioral health and functioning across six subscales: Depression/functioning, interpersonal relationships, psychosis, substance abuse, emotional lability, and self-harm. While there are no cutoff scores or quartiles used to categorize patients using BASIS-24 scores in the literature, having higher scores likely indicates worse mental well-being and functioning within a clinical patient population [27]. Thus, we categorized participants scoring at or above the overall BASIS-24 score median as having worse overall mental health well-being and functioning or a high behavioral health symptoms score (*n* = 264) and those scoring below the median as having better overall mental health well-being and functioning or a low behavioral health symptoms score (*n* = 263). As psychotic disorders partially comprise serious mental illnesses, we additionally ran analyses using the BASIS-24 psychosis subscale median as the cutoff score and found similar results for primary and secondary outcomes (data not shown). We chose the analyses using the overall BASIS-24 score as the cutoff with the understanding that smoking abstinence outcomes for participants with high BASIS-24 scores may reasonably reflect outcomes for patients with serious mental health challenges.

We examined 30-day self-reported smoking abstinence at 6-months as our primary outcome. Non-respondents at 6 months were treated as people who smoke. We had three secondary outcomes: engagement with cessation treatment at 6 months, which included receipt of telephone counseling, self-reported use of cessation medications, and a 24-h quit attempt. For secondary outcomes, we treated non-respondents as not having engaged with cessation treatment at 6 months (i.e., no telephone counseling, not actively using cessation medications, and no ≥ 24 h quit attempts made).

We compared demographic, health, and smoking characteristics at baseline between the two groups, defined as high vs. low scores of behavioral health symptoms. Wilcoxon’s rank-sum test was used for continuous variables and Chi-square or Fisher’s exact test for categorical variables. Next, logistic regression was used to compare groups on primary and secondary outcomes, adjusting for baseline cigarettes per day and clustering by site. For each outcome, we tested for an interaction effect between treatment assignment and behavioral health symptoms score category (high vs. low). Finally, we performed multivariable logistic regression to determine the factors associated with high behavioral health symptoms and functioning scores, and report findings as adjusted odds ratios (AOR) with 95% confidence intervals (CIs). All statistical tests were 2 sided with *P* < 0.05 indicating statistical significance. Analyses were performed with SAS, version 9.4 (SAS Institute Inc).

## Results

We describe demographic, health, and smoking characteristics by behavioral health symptoms score category at baseline in Table 1 (see end of document). Participants with high vs. low scores of behavioral health symptoms were similar in most demographic characteristics. The study population across both groups was predominantly male, mostly Black or white, and not Hispanic/Latino, with a mean age of 53 years old. In both groups, participants smoked an average of 16 cigarettes per day and were “very motivated” to quit smoking. However, compared to those with low behavioral health symptoms scores (i.e., better mental well-being and functioning), those with high scores were significantly more likely to have heavier nicotine dependence (% reporting smoking within 5 min of waking in the morning; 40% vs. 27%), were more likely to report current sedative/sleeping pill use (18% vs. 8%, *p* < 0.01) and current antidepressant use (53% vs. 35%, *p* < 0.0001).

**Table 1:** Baseline characteristics in participants with high vs. low behavioral health symptoms scores

Baseline characteristic	Low (*N* = 263)	Low %	High (*N* = 264)	High %	*p* value*
**Age in years, mean**	53.8^a^	-	53.3^b^	-	0.49
**Male gender**	249	95	238	90	
**Race**					0.46
American Indian/Alaska Native/Asian	6	2	4	2	
White	134	51	140	53	
Black or African American	97	37	83	31	
More than one race	13	5	17	6	
Missing	13	5	20	8	
**Hispanic ethnicity**					**0.05**
Hispanic or Latino	40	15	58	22	
Not Hispanic or Latino	222	84	205	78	
**Education**					0.19
High school grad or less	106	40	115	44	
Associate’s degree/Some college	115	44	121	46	
4-year college graduate or higher	42	16	28	11	
**Marital status**					0.70
Married/living with partner	79	30	77	29	
Separated/divorced/widowed	112	43	122	46	
Never married	71	27	65	25	
**Cigarettes per day, mean**					
How many cigarettes do you smoke per day?	15.5^c^		17.1^d^		0.09
**Time to first cigarette**					** < 0.01**
Within 5 min	72	27	106	40	
6–30 min	88	33	89	34	
31–60 min	46	17	27	10	
After 60 min	55	21	41	16	
**Quit attempt in last year**	154	59	152	58	0.82
**Plan to quit smoking**					0.21
Within the next 30 days	188	71	177	67	
Not within the next 30 days	72	27	86	33	
**Motivation to quit**					0.08
Not at all motivated	4	2	1	0	
Just a little motivated	15	6	29	11	
Somewhat motivated	86	33	87	33	
Very motivated	158	60	146	55	
**Current alcohol/substance use**					
Alcohol	74	28	86	33	0.27
Cannabis	16	6	28	11	0.06
Cocaine	0	0	3	1	0.25
Amphetamine stimulates	1	0	2	1	0.99
Sedatives/sleeping pills	20	8	47	18	** < 0.01**
Opioids	3	1	9	3	0.14
**Past/current use of bupropion**	43	16	49	19	0.50
**Past/current use of NRT**	122	46	136	52	0.24
**Current use of antidepressants**	92	35	140	53	** < .0001**
**Degree of support of others for quitting**					**0.03**
Not at all supportive	23	9	38	14	
A little supportive	24	9	13	5	
Somewhat supportive	49	19	61	23	
Very supportive	164	62	150	57	

*NRT* Nicotine Replacement Therapy

^*^We used Wilcoxon’s rank-sum test for continuous variables and Chi-square or Fisher’s exact test for categorical variables

Standard Deviations: ^a^12, ^b^11, ^c^11, ^d^12

At 6 months, participants with low behavioral health symptoms scores reported long-term abstinence rates that were not significantly different from those with high behavioral health symptoms scores (20% vs 13%), as shown in Table 2. However, we found an interaction effect between behavioral health symptoms scores and treatment assignment. At 6 months, participants with low behavioral health symptoms scores in the specialized, multi-session telephone smoking cessation program were more likely to report 30-day abstinence compared to participants with low behavioral health symptoms scores referred to a state Quitline (26% vs 13%, OR = 2.3, 95% CI = 1.8, 2.9). Participants with low behavioral health symptoms scores in the intervention arm were also more likely to have made a quit attempt longer than 24 h at 6 months compared to those who received the state Quitline referral (62% vs 49%, OR = 1.7, 95% CI = 1.2, 2.5) (Table 3).

**Table 2:** Self-reported abstinence at 6 months in participants with high vs. low behavioral health symptoms scores

	**Specialized n/N**	**Specialized %**	**Quitline n/N**	**Quitline %**	**OR**	**95% CI**	*p*-value*
**Behavioral health symptoms scores**							**0.03**
Low	32/125	26	18/138	13	2.3	1.8, 2.9	** < 0.01**
High	15/121	12	18/143	13	1.1	0.6, 2.0	0.79

*CI* Confidence Interval, *OR* Odds Ratio (adjusting for baseline cigarettes per day and site clustering)

^*^P value for interaction term. Participants were considered abstinent at 6-month follow-up if they reported not having smoked any cigarettes in the prior 30 days. We categorized participants as high vs. low behavioral health symptoms scores based on whether they were below or above the median score on the BASIS-24 subscale at baseline

**Table 3:** Cessation treatment use at 6 months in participants with high vs. low behavioral health symptoms scores

	**Specialized n/N**	**Specialized %**	**Quitline n/N**	**Quitline %**	**OR**	**95% CI**	*p* value*
**Telephone counseling**							0.81
Low	60/125	48	36/138	26	2.7	0.8, 9.3	0.10
High	62/121	51	47/143	33	2.3	1.3, 4.3	**0.01**
**NRT/buproprion**							0.19
Low	73/125	58	71/138	51	1.3	1.0, 1.7	0.05
High	71/121	59	87/143	61	0.9	0.5, 1.6	0.73
**Quit attempt > 24 h**							**0.03**
Low	78/125	62	68/138	49	1.7	1.2, 2.5	**0.01**
High	66/121	55	85/143	59	0.9	0.6, 1.3	0.35

*CI* Confidence Interval, *OR* Odds Ratio (adjusting for baseline cigarettes per day and site clustering)

^*^P-value for interaction term. Participants were considered abstinent at 6-month follow-up if they reported not having smoked any cigarettes in the prior 30 days. NRT = nicotine replacement therapy. We categorized participants as high vs. low behavioral health symptoms scores based on whether they were below or above the median score on the BASIS-24 at baseline

By contrast, we observed no significant difference in 30-day abstinence among participants with high behavioral health symptoms scores between the treatment groups at 6 months (12% vs. 13%, OR = 1.1, 95% CI = 0.6, 2.0). There was also no difference between treatment arms among people with high behavioral health symptoms scores in the percent of individuals making at least one ≥ 24 h quit attempts at 6 months (56% vs. 59%, OR = 0.9, 95% CI = 0.6,1.3). Notably, people with high behavioral health symptoms scores in the intervention arm made greater use of telephone counseling than those in the control arm (51% vs. 33%, OR = 2.3, 95% CI [1.3, 4.3]. We did not observe an interaction effect in the relationship between treatment condition and use of telephone counseling or NRT/bupropion at 6 months (Table 3).

On multivariable analysis (Table 4), we found that Hispanic/Latino ethnicity (OR 1.7, 95% CI [1.0, 2.7]), current sedative/sleeping pill use (OR 2.1, 95% CI [1.1, 3.7]), current cannabis use (OR 2.0, 95% CI [1.0, 4.1]), and current antidepressant use (OR 1.7, 95% CI [1.2, 2.5]) were independently associated with having high behavioral health symptoms and functioning scores. In general, a greater degree of support from others for quitting was associated with lower odds of having high behavioral health symptoms and functioning scores (Not at all supportive as referent; A little supportive OR 0.3 [0.1, 0.8]; Somewhat supportive OR 0.9 [0.4, 1.7]; Very supportive OR 0.5 [0.3, 1.0]). Similarly, a greater time to first cigarette upon waking was associated with lower odds of having high behavioral health symptoms and functioning scores (Within 5 min considered referent; 6–30 min OR 0.7 [0.5, 1.2]; 31–60 min OR 0.4 [0.2, 0.8]; After 60 min OR 0.6 [0.3, 1]).

**Table 4:** Multivariable analysis of baseline characteristics associated with high behavioral health symptoms scores

	**OR**	**95% CI**	***p* value**
**Gender (reference is male)**	1.4	0.7, 2.8	0.38
**Hispanic ethnicity**			
Not Hispanic or Latinx	Reference		
Hispanic or Latinx	1.7	1.0, 2.7	**0.04**
**Cigarettes per day**	1.0	1.0, 1.0	0.56
**Time to first cigarette**			
Within 5 min	Reference		
6–30 min	0.7	0.5, 1.2	0.20
31–60 min	0.4	0.2, 0.8	**0.01**
After 60 min	0.6	0.3, 1.0	0.07
**Current alcohol/substance use**			
Sedatives/sleeping pills	2.1	1.1, 3.7	**0.02**
Cannabis	2.0	1.0, 4.1	**0.05**
**Current use of antidepressants**	1.7	1.2, 2.5	**0.01**
**Degree of support of others for quitting**			
Not at all supportive	Reference		
A little supportive	0.3	0.1, 0.8	**0.02**
Somewhat supportive	0.9	0.4, 1.7	0.67
Very supportive	0.5	0.3, 1.0	**0.05**

*CI* Confidence Interval, *OR* Odds Ratio

## Discussion

This study examines the effectiveness of an intensive telephone intervention for smoking cessation compared to referral to the state Quitline among mental health patients who smoke referred by mental health providers at six VA facilities. We found similarities in many demographic characteristics at baseline, including self-reported race, ethnicity, income, educational attainment, motivation to quit, nor number of cigarettes smoked per day. Regarding differences, people with high behavioral health symptoms scores reported heavier nicotine dependence and were more likely to report current use of sedatives, sleeping pills, and/or antidepressants at baseline. We found a significant interaction effect between behavioral health symptoms scores and treatment assignment. People with low behavioral health symptoms scores in the specialized counseling arm were significantly more likely to report 30-day abstinence at 6 months and to have made a quit attempt compared to people with low behavioral health symptoms scores in the state Quitline counseling arm. People with high behavioral health symptoms scores did not have significantly different abstinence outcomes and did not differ in the likelihood of having made a quit attempt at 6 months based on treatment assignment.

The significant interaction effect between behavioral health symptoms score level and treatment assignment has several implications. It adds nuance to existing research that suggests mental health patients benefit from intensive, specialized telephone interventions [21, 22]. In our study, we found that people who smoke with low behavioral health symptoms scores benefited significantly more from an approach that included multiple sessions, relapse-sensitive timing, and customization for mental health patients. People with high behavioral health symptoms scores did not demonstrate higher cessation rates with this intensive approach. Thus, a multivariable analysis was performed to identify barriers to smoking cessation in this cohort. Higher behavioral health symptoms and functioning scores were independently associated with Hispanic/Latino ethnicity, as well as current antidepressant, cannabis, and/or sedative/sleeping pill use. In addition, lower social support for quitting and shorter time to first cigarette, which suggests higher nicotine addiction, were associated with higher odds of having high behavioral health symptoms. This suggests people who smoke with high behavioral health symptoms scores may have had cessation needs that were not addressed by the specialized counseling program and thus need a different approach that accounts for use of non-tobacco substances, lower degrees of social support, and heavier nicotine dependence. Additionally, the specialized counseling approach may not have been sufficient to address more severe behavioral health symptoms. Integrated care, which involves delivering treatment for tobacco use and psychiatric care in a single clinical setting, has been found to be effective for patients with PTSD and may be a model for people who smoke with high behavioral health symptoms scores [28]. Finally, the relationship between Hispanic/Latino ethnicity and higher behavioral health symptoms is not clear and may warrant future research. Regarding secondary outcomes, it is notable that the participants with high behavioral health symptoms and functioning scores did engage in telephone counseling at significantly higher rates in the intervention group compared to those in the control group. This suggests that they have significant motivation to quit and perhaps responded favorably to certain qualities of the intervention, such as VA-based delivery or consideration of comorbid mental health diagnoses.

There are several limitations to consider. A secondary data analysis is by nature limited by the data collection from the parent study, which relied on self-reported abstinence. BASIS-24 is well-regarded as a measure of behavioral health symptoms and functioning, but a cutoff point for defining categorically a high behavioral health symptoms score has not been validated in the literature. Thus, our use of the overall BASIS-24 score median as a cutoff score to compare patient groups by their level of mental well-being and functioning requires further evaluation longitudinally, and in other clinical and research settings. In addition, only approximately 10% of eligible patients were referred to the parent study, thus raising the question of selection bias among referring providers in favor of people whom they may perceive to be more likely to quit. Finally, the study examined a veteran population that was overwhelmingly male, so the results may not be generalizable to non-veterans and/or females.

## Conclusions

Further research to explain different smoking cessation patterns between people with low behavioral health symptoms scores and high behavioral health symptoms scores is needed. Qualitative evidence may provide further insight into what aspects of the specialized intervention were particularly beneficial for people who smoke with better mental well-being and functioning and less efficacious for people who smoke with worse mental well-being and functioning. Understanding the nuances in cessation patterns among people who smoke with mental health diagnoses will inform cost-effective, evidence-based public health decisions.

## Data Availability

The datasets used and/or analyzed during the current study are available from the corresponding author on reasonable request.

## Abbreviations

BASIS-24: 24-Item Behavior and Symptom Identification Scale
OR: Odds ratio
CI: Confidence interval
SMI: Serious mental illness
PTSD: Post-traumatic stress disorder
VA: Veterans Association
EMR: Electronic medical record
NRT: Nicotine replacement therapy
